# Cancer of Female Breast

**Published:** 1873-11

**Authors:** 


*OBSTETRICAL JOURNAL OF GREAT BRITAIN AND IRELAND.*

Cancer of the Female Breast.—In the course of an interesting analysis of 295 cases of cancer of the female breast, in a paper by Dr. John C. Peters, read before the Medical Library and Journal Association of New York, the propriety of operating is thus considered:

"There are some who say, never operate. I think this opinion comes from the older members of the profession, who are inclined to look beyond the simple performance of the operation. The younger men, many of them, say, operate. Upon this question, I, perhaps, can do no better than to refer you to the opinions of two men who are among the most experienced of the profession, and who have had abundant facilities for making observations. I refer to Paget and Sibley, both of London.

"Mr. Paget has shown, from his statistics, that the average length of life after an operation is 43 months; and that the average length of life without an operation is 55 months. Mr. Sibley has shown, from his statistics, that the average length of life after an operation is 53 months, and that the average length of life without an operation is 32¼ months. Here are the results of observations made by two distinguished authorities. I think all that I am justified in saying upon this point is, that every case must be taken by itself, looked at with all its surroundings, before a decision is given for or against an operation.

"The older the patient, other things being equal, the more favorable for operation. If the cancer has extended so that we have secondary cancer, it is not surgical to operate. Therefore, when the axillary glands or the skin is involved, and we have the local and constitutional disease both existing, I regard it as unfavorable for an operation. When the tumor is isolated, and there are no secondary manifestations, the conditions are favorable for an operation, and the sooner it is performed, the better the chance of preserving the life of the patient. If the patient comes complaining of an irritable tumor of the breast, apparently accompanied with some disorder of menstruation, I should recommend, first, careful attention to the general health; and, second, its removal at once if it is found to be increasing in size. There is another condition in which I would operate, and that is in the sloughing cases. Then it is done simply to make the patient more comfortable. Practically speaking, these cases do not belong to secondary cancer, and the operations are not unfavorable. But with all these cases, we must use our own discretion. Select the cases and give them the benefits and advantages of an operation."

The conclusions to which Dr. Parker has arrived are chiefly as follows:

1st. That the disease is not hereditary, or, if so, only in a very limited degree.

2d. That the disease begins as a local disease, positively and purely. It becomes constitutional just as syphilis begins a local disease and becomes constitutional.

3d. That the disease occurs in those of vigorous health, instead of being connected with those conditions in which consumption occurs.

4th. That cancerous parents may beget tuberculous offspring. That is, feeble constitutions arising from the effects of cancer will not beget cancer, but the diseases which follow in their line are tuberculous.

5th. That the moral condition has a powerful influence on the development or the prevention of the development of cancer.

6th. The parallelism and analogy existing between cancer and syphilis is very striking.

